# Intimate partner violence, anxiety, stress, and depression among Israeli Arab married women in the shadow of the second wave of the COVID-19 pandemic

**DOI:** 10.1371/journal.pgph.0006670

**Published:** 2026-06-30

**Authors:** Ola Ali-Saleh, Inbal Halevi Hochwald, Jalal Tarabeia, Fuad Basis

**Affiliations:** 1 Department of Nursing, The Max Stern Yezreel Valley College, Emek Yezreel, Israel; 2 The Research Authority, Saint Vincent de Paul Hospital, Nazareth, Israel; 3 Faculty of Medicine, Technion – Israel Institute of Technology, Haifa, Israel; 4 Emergency Medicine, Rambam Health Care Campus, Haifa, Israel; University of Manitoba College of Medicine: University of Manitoba Max Rady College of Medicine, CANADA

## Abstract

The COVID-19 pandemic was accompanied by lengthy lockdowns, school closures, and mandatory stay-at-home orders, causing stress, anxiety and depression. Since most Arabs in Israel live in closed communities in villages, with high birthrates and specific demographic characteristics, during the pandemic they may have experienced higher rates of stress, anxiety, depression, and violence against women compared to the general population. To measure the rate of stress, anxiety, and depression among Arab women during the second wave of the COVID-19 pandemic and to attempt to build a model that links these parameters and intimate partner violence (IPV). 602 women were recruited from Israeli Arab society through an online self-reported questionnaire. All participants were involved in an intimate relationship; the vast majority were married. Arab women experienced more anxiety, depression, and a higher rate of spousal violence than the general population. Violence was more prevalent among religious than among secular Muslim women and more frequent among Muslim women than among Christian women. A high rate of childbirth, lower education, and low socioeconomic background were highly correlated with violence. Living in close communities and fear of shaming led to a lower rate of seeking intervention. Authorities should be aware of different minorities` needs during pandemics and deliver suitable and discrete interventions. Furthermore, intervention in the education of minority groups, especially religious Muslim women, may lead to lower childbirth rates and improved socioeconomic status, and thus may decrease the prevalence of violence against women during stressful prolonged situations.

## Introduction

Intimate partner violence (IPV) is a serious public health issue. The lifetime prevalence of IPV among women reportedly ranges from 13% to 61% worldwide [1]. The 2011 National Intimate Partner and Sexual Violence Survey (NISVS) in the United States revealed that more than one in five women (22.3%) and nearly one in seven men (14.0%) experienced severe physical violence by an intimate partner at some point in their adult lives [2].

IPV involves a variety of aggressive actions, such as physical, psychological, economic, or sexual harassment [3]. Men and women of all social strata may experience IPV. However, the incidence of IPV is higher among women and certain ethnic groups [4]. The factors associated with IPV are unemployment [5], low income, low education [6], economic dependence [7], spousal unemployment [8], and stress [9].

In Israel, the IPV rate was found to be high among Arabs. In addition, some of the abovementioned common risk factors related to IPV are common in the sociodemographic characteristics of Arab society [10]. Furthermore, an association was found between Arab women’s IPV and postpartum depression [3].

The Israeli Arab minority comprises 21% of the total state’s population. Approximately 80% are Muslims, approximately 50% of whom live in small villages in the northern part of the country and are of lower socioeconomic status. Only 34% of Arab women belong to the workforce, whereas 52% of Jewish women do [11]. During the second COVID-19 outbreak, there was a rise in violence and criminal activity among Arabs, and 40% of all murder cases in the country occurred in the Arab sector, i.e., twice their percentage in the country’s population. Sixty-five percent of these cases involved women murdered by their intimate partner [12].

The second wave of the COVID-19 pandemic took place from mid-June 2020 until the end of October 2020. The incidence of new confirmed cases of COVID-19 has increased to 4000–6000 new cases a day. The number of deaths reached approximately 1200 (130 deaths per million). Until the end of October, nearly 180,000 confirmed cases were reported in Israel, 50,000 of which were active [13]. Since the first outbreak, the government has imposed an emergency lockdown with restrictions on public transportation, gatherings in wedding halls, and prayer places. This led to a reduction of 30% of employees in the public sector [13,14]. The quarantine and job loss led to economic instability in Arab society, which previously had a lower income than average. On the other hand, the traditional pattern of Arabs living in villages and small communities has led to higher infection rates and mortality rates [15,16].

In Israel, as in other countries, school closures, social isolation, and job loss contribute to increased psychosocial stress, particularly among families with pre-existing vulnerability factors [17]. Given the relationship between psychosocial stress and IPV, the prevalence of reported IPV and calls to emergency support lines during lockdowns increased significantly with the progression of the pandemic [18–20]. Among the Arab female population in Israel, the rate of reported mental health issues, such as anxiety and depression, increased significantly during the COVID-19 pandemic [21,22]. The hotline available for Arab women in the city of Nazareth (an Arab city) dealing with ‘physical and sexual assault’ from intimate partners increased by 200% compared with the first lockdown (11 March 2020) compared to the same period in 2019 [23].

### Objectives

To measure the rates of stress, anxiety, and depression among Arab women during the second wave of the COVID-19 pandemic and to build a model that links these parameters with IPV.

## Method

### Ethics statement

The study was approved by the Max Stern Academic College of Emek Yezreel Helsinki Committee in accordance with the declaration of Helsinki (approval YVC-EMEK 2020–91).

A link to an electronic questionnaire, accompanied by a short introduction, was distributed anonymously through social media platforms, mainly Facebook and WhatsApp, between September 30, 2020 and October 10, 2020. The invitation described the study as examining Arab women’s experiences, intimate relationships, and mental health during the COVID-19 pandemic, rather than focusing solely on IPV. A snowball sampling approach was used, and participants were asked to forward the questionnaire link to other eligible women in their social networks.“. Participants consent to participate was provided by agreeing to answer the questionnaire. The inclusion criterion was being an Arab woman over 18 years old who is engaged in an intimate relationship. The questionnaire was developed in the Arabic language and was distributed after the second wave of the COVID-19 pandemic. Sociodemographic data (age, marital status, number of children, education, religion, residency, employment, and income) were collected. Income was assessed as self-reported household income relative to the average household income in Israel in 2020, and place of residence was assessed by asking participants whether they lived in an urban locality/city or in a rural locality/village. In addition, intimate partner violence-related variables (physical, sexual, emotional, and economic violence) were assessed via 10 items about violence perpetrated by the participant’s spouse (categories: 1. never, 2. rarely, 3. often, and 4. always). The questions were those used by the Israeli Ministry of Health, which is based on the IPV United States (US) Prevention Services Task Force, 2004, and research conducted in Israel [24]. A woman was defined as a victim of violence if she answered affirmatively to at least one of ten questions (in the present sample, Cronbach’s α was.80). Items 6, 9, and 10 dealt with physical violence (Cronbach’s α = .69); Items 1, 2, 7, and 8 dealt with emotional or verbal violence (Cronbach’s α = .53); and Items 3, 4, and 5 dealt with social or economic violence (Cronbach’s α = .63). Social/economic violence included items assessing attempts by the partner to isolate the woman from family and friends, requiring financial dependence on the partner for daily expenditures, and extreme jealousy expressed through controlling behaviors such as stalking, frequent calling, or demanding to know the woman’s whereabouts. These items were intended to capture coercive control that restricts women’s social autonomy and financial independence. Total scores were computed with item means.

When dealing with mental health-related variables, anxiety was evaluated by a generalized anxiety disorder (GAD-7) questionnaire that proposed a 7-item scale related to anxiety, developed by Spitzer et al. [25], α = .93. Subjective anxiety symptoms during the past month were reported on a Likert scale ranging from 0 = never to 3 = almost every day. The combined score for the entire questionnaire ranged from 0--21 and was computed as a categorical variable defined as minor anxiety (0--4 points), mild anxiety (5--9 points), moderate anxiety (10--14 points), or severe anxiety (scores above 15 points).

The Patient Health Questionnaire-2 (PHQ-2) developed by Kroenke et al. [26] was used to evaluate depression. The current study questionnaire consisted of two items from the PHQ-2. Both items measure depressed mood and loss of interest in previously pleasurable activities (anhedonia). The presence of a single item is an indicator of suspected major depressive disorder (MDD) for any depressive disorder according to the DSM-IV. PHQ-2 scores can range from 0 to 6, and a cut-off point ≥3 indicates significant clinical depression. The correlation coefficient between the two items in the current study was r = .75 (p < .001).

The stress level was evaluated via the Perceived Stress Scale (PSS), which consists of 10 items (Cohen & Williamson; Cronbach’s α = 0.86) [27]. The scale measures perceptions and feelings related to general stress levels in recent years on a scale ranging from 0 = never to 4 = often. The overall score of the ten items ranges from zero to 40, with higher scores indicating higher levels of perceived stress. Scores between 0 and 13 were considered minor stress levels, scores between 14 and 26 were considered moderate stress levels, and scores ranging between 27 and 40 were considered high stress levels.

The data were analyzed via SPSS 28 software (IBM Corporation, Armonk, NY, USA). Descriptive statistics were conducted to obtain an overview of the participant’s background and clinical classification characteristics. Cronbach’s alpha was used for internal consistency reliability, and scale scores were computed from item means or sums. Owing to positively skewed distributions for the violence variables, they were log-transformed. Pearson correlations were calculated to evaluate the links between all the study variables. Relationships between the study variables and major demographic characteristics were assessed with Pearson and Spearman correlations, as well as with a series of independent t-tests. By the research aims, multiple linear regression analysis models were calculated for the study variables. The first step included demographic characteristics, whereas the second step included the study variables, which were added incrementally according to the study model. Finally, PROCESS modelling, as outlined by Hayes et al. [28], was applied, and a bootstrapping method (5,000 bootstrapping samples) was used, as was a 95% bias-corrected confidence interval for the effect size estimates. The number of children, religion, religiosity, and income were controlled. Four models were applied, with the total score for intimate partner violence and the three subscales as independent variables, anxiety and stress as the mediators, and depression as the dependent variable. Statistical significance was set at *p* < 0.05.

The subjects were provided with authentic information about the goals of the research. Those who were interested in voluntarily participating in the research signed an electronic informed consent form attached to the introduction before completing the questionnaire. The participants were guaranteed that they could leave the research at any time, that their answers would be kept discrete, and that questionnaire data would be collected and analyzed anonymously.

## Results

602 Israeli Arab women participated in an online self-report questionnaire. All the participants were engaged in intimate relationships; the vast majority were married (96%). They were aged between 18 and 44 years, and 92% of the women were between 25 and 44 years old, with up to six children (mean = 1.81, SD = 1.31) (Table 1). Slightly more than half of the participants were Muslim (56%), and the others were Christian (44%), very religious (approximately 35%), or partly religious (49%). A little over half were urban (55%). Academic degree education was reported by 77% of the women and 49% of their partners. Most participants were employed (78% of the women and 82% of their partners), but to a lesser extent during the pandemic (approximately 36% of the women and 25% of their partners), where at least one partner was on unpaid leave in approximately 43% of the couples. Income was average or above average in approximately half of the cases (Table 1).

**Table 1:** Participants’ sociodemographic characteristics (*N* = 602).

Characteristics	%	*N*
**Age, years**		
18-24	8.3	50
25-34	59.8	360
35-44	31.9	192
**Mean number of children (SD** ^**a**^ **), range**	1.81 (1.31), 0-6	
**Religion**		
Muslim	55.6	335
Christian	44.4	267
**Religiosity**		
Secular	16.4	99
Partly religious	49.0	295
Religious	34.6	208
Residence		
Urban	55.0	331
Rural	45.0	271
Woman’s Education		
Less than academic	23.1	139
Academic	76.9	463
Partner’s education		
Less than academic	51.3	309
Academic	48.7	293
Woman works outside the home	78.4	472
Woman works outside the home during the pandemic,	36.2	218
Partner works outside the home	82.1	494
Partner works outside the home during the pandemic	24.8	149
One partner is on unpaid leave	43.5	262
Income		
A lot below average	20.3	122
Somewhat below average	29.7	179
Average	32.9	198
Above average	17.1	103

^a^SD, standard deviation

High incidences of intimate partner violence, anxiety, stress, and depression were reported, as shown in Table 2. Approximately 70% (424/602) of the participants reported any intimate partner violence. Emotional or verbal violence was the leading reported type of violence (approximately 57%, 341/602), followed by social or economic violence (45.3%, 273/602) and physical or sexual violence (20.8%; 125/602). Approximately 58% (351/602) of them were classified as having moderate to severe levels of anxiety, and approximately 77% (466/602) of them were classified as having moderate to high levels of stress. In addition, approximately 56% (340/602) of the participants were classified as suspected of having major depression (Table 2).

**Table 2:** Classification of intimate partner violence, anxiety, stress, and depression (*N* = 602).

Variables	%	*N*
Total intimate partner violence	70.4	424
Emotional or verbal violence	56.6	341
Physical or sexual violence	20.8	125
Social or economic violence	45.3	273
**Anxiety**		
None	15.0	90
Minor	26.7	161
Moderate	31.6	190
Severe	26.7	161
**Stress**		
Minor	22.6	136
Moderate	61.1	368
High	16.3	98
**Depression**	56.5	340

Overall, approximately 91% of the women were positive for at least one domain of the mental health-related variable (MHRV) (*n* = 548), and approximately 82% were positive for anxiety, stress, or depression (*n* = 496). Approximately 74% of them reported comorbidity of any two characteristics, including violence (*n* = 448), and approximately 63% reported comorbidity for anxiety, stress, or suspected major depression (*n* = 379).

Table 3 presents the distributions of the study variables and their Spearman intercorrelations. The means and standard deviations for intimate partner violence were rather low, and the means for anxiety (M = 10.9), stress (M = 19.12), and depression (M = 2.99) were moderate. All the correlations are positive and significant, indicating that greater intimate partner violence, greater anxiety, greater stress, and greater depression are interrelated.

**Table 3:** Means, standard deviations, and Spearman’s correlations for the study variables (*N* = 602).

	Variables	M (SD)	2.	3.	4.	5.	6.	7.
1.	**Total intimate partner violence (0–3)**	0.31 (0.42)	.89***	.76***	.85***	.42***	.32***	.36***
2.	**Emotional/verbal violence (0–3)**	0.37 (0.47)		.55***	.58***	.42***	.34***	.39***
3.	**Physical/sexual violence (0–3)**	0.15 (0.38)			.52***	.29***	.19***	.20***
4.	**Social/economic violence (0–3)**	0.37 (0.57)				.31***	.23***	.26***
5.	**Anxiety (0–21)**	10.90 (5.76)					.72***	.80***
6.	**PSS stress (0–40)**	19.12 (7.39)						.66***
7.	**Depression (0–6)**	2.99 (1.82)						

***p < .0001

The relationships between the study variables and the demographic characteristics presented in Table 1 were assessed. The results demonstrated several relationships: living with children at home was positively related to IPV (total score: r = .14, *p* < .001; dimensions: r = .11 *p* = .008 to r = .16 *p* < .001), which means that women who cared for more children reported a higher level of IPV. The regression distributions revealed significant differences (Table 4). Compared with Christian women, Muslim women reported greater IPV (M = 0.33, SD = 0.41 vs. M = 0.27, SD = 0.44, t(600) = 2.38, *p* = .018), higher anxiety scores (M = 11.97, SD = 5.59 vs. M = 9.55, SD = 5.71, t(600) = 5.21, *p* < .001), higher stress scores (M = 19.84, SD = 7.33 vs. M = 18.20, SD = 7.37, t(600) = 2.72, *p* = .007) and greater depression scores (M = 3.27, SD = 1.81 vs. M = 2.64, SD = 1.78, t(600) = 4.30, *p* < .001). The results by religious status revealed that religious women reported higher anxiety scores than secular or partly religious women did (M = 11.90, SD = 5.58 vs. M = 10.38, SD = 5.77, t(600) = 3.10, *p* = .002), higher stress scores (M = 20.00, SD = 7.46 vs. M = 18.64, SD = 7.21, t(600) = 2.14, *p* = .030), and greater depression scores (M = 3.21, SD = 1.81 vs. M = 2.88, SD = 1.81, t(600) = 2.15, *p* = .032). A high-income level was correlated with lower levels of IPV (total score: r = -.23, *p* < .001; dimensions: r = -.16 to r = -.20 *p* < .001), lower anxiety scores (r = -.24, *p* < .001), stress scores (r = -.12, *p* = .003), and depression scores (r = -.16, p < .001).

**Table 4:** Multiple regression models for intimate partner violence (IPV), anxiety, stress, and depression (*N* = 602).

	IPV (total score)	Anxiety	Stress	Depression
	β (SE)	β (SE)	β (SE)	β (SE)
**Number of children**	.14 (0.01)***	-.02 (0.16)	-.07 (0.22)	.01 (0.03)
**Religion (Muslim)**	.04 (0.02)	.14 (0.44)***	.07 (0.61)	.02 (0.09)
**secular or partially religious**	.03 (0.02)	-.05 (0.46)	-.05 (0.63)	.01 (0.10)
**Income (higher)**	-.24 (0.01)***	-.12 (0.20)**	-.03 (0.28)	.03 (0.04)
**IPV (total score)**	---	.38 (0.84)***	.31 (1.14)***	.03 (0.19)
**Anxiety**	---	---	---	.66 (0.01)***
**Stress**	---	---	---	.18 (0.01)***
**Adj. R** ^**2**^	.07***	.22***	.11***	.65***

*p < .05, **p < .01, ***p < .001, SE, standard error

It should be noted that most Christian women were partly religious (56.9%) or secular (23.2%), while most Muslim women were religious (46.3%) or partly religious (42.7%) (p < .001). Taken together, the differences in IPV, anxiety, stress, and depression, by the interaction between religion and religious status, were not significant (p = .188 to p = .904). That is, the general pattern of differences by religion, or differences by religious status, was not different by sub-group.

The study hypotheses were examined while controlling for the number of children, religion (1-Muslim, 0-Christian), religiosity (1-secular or partly religious, 0-religious), and income level (1-a lot below average, 5-a lot above average). Notably, the distribution of income level did not deviate from normality (skewness = 0.26, SE = 0.10) and was thus considered continuous. Furthermore, the number of children was highly related to the categories of age (r_s_ = .56, *p* < .001); thus, controlling for the number of children partly controls for age as well. In addition, higher income was significantly related to academic education (for the woman: r = .24, *p* < .001; for her partner: r = .34, *p* < .001), such that controlling for income partly controls for academic education as well.

To examine the predictors of sociodemographic characteristics, anxiety, stress, and depression associated with intimate partner violence (IPV), four multiple regression models were applied to assess the extent to which IPV, anxiety, stress, and depression are related to demographic characteristics and each other. The first step in all the models included the demographic characteristics, whereas the second step included the study variables, which were added incrementally, according to the study model. The results shown in Table 4 demonstrate that all four models are significant.

Seven percent of the variance in IPV was explained by demographic characteristics (i.e., caring for more children and coping with lower levels of income). Twenty-two percent of the variance in anxiety was explained by demographic characteristics and IPV. Being a Muslim woman, coping with lower levels of income, and suffering from higher IPV were related to higher levels of anxiety. The next 11% of the variance in stress was explained by the study variables. In addition to demographic characteristics, higher levels of IPV were related to greater stress. Finally, 65% of the variance in depression was explained, such that in addition to demographic characteristics, higher levels of anxiety and stress were related to higher levels of depression.

Differences in IPV (stress, anxiety, and depression as well) by type of residence (rural/urban) were not significant. Adding the interactions with income, number of children per household, and level of religiousness, showed that all interactions were not significant. That is, differences in the dependent variables were significant by income, number of children per household, and level of religiousness, but were not significant by type of residence (rural/urban) or by income, number of children per household, and level of religiousness, within type of residence. As all these differences were not significant, we chose not to add these results to the manuscript.

**Fig 1:**
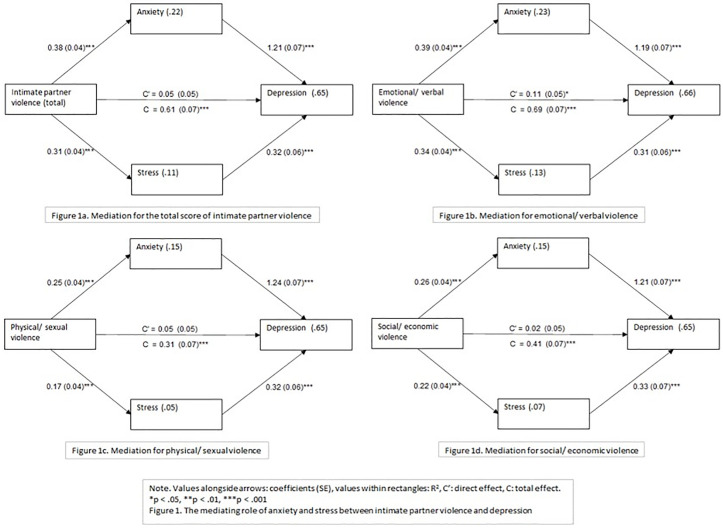
The mediating role of anxiety and stress between intimate partner violence and depression. **a**. Mediation for the total score of intimate partner violence, **b**. Mediation for emotional/ verbal violence, **c**. Mediation for physical/ sexual violence, **d**. Mediation for social/ economic violence. Note: Values alongside arrows: coefficients (SE), values within rectangles: R^2^; C’: direct effect; C: total effect. *p < .05, **p < 0.01, ***p < 0.001.

The mediation model was applied via the PROCESS procedure (Hayes [29], Model 4 for parallel mediation. Continuous variables were standardized, and the number of children, religion, religious status, and income were controlled for. Four models were calculated, with the total score for IPV and the three subscales as independent variables, anxiety and stress as the mediators, and depression as the dependent variable (Fig 1). All the indirect paths were found to be significant, such that higher levels of IPV were related to higher levels of anxiety and stress, which were then related to higher levels of depression.

## Discussion and conclusions

The global coronavirus disease 2019 (COVID-19) outbreak occurred early in 2020, erupted rapidly, and was associated with stress and panic related to the uncertainty of disease severity, lack of proven treatment, absence of available effective vaccines and lengthy lockdowns [13,30]. Social distancing and isolation are central strategies adopted by many countries and settings to protect public health. Social isolation requires families to remain in their homes, resulting in intense and unrelieved contact as well as the depletion of existing support networks [31**–**33].

The current study revealed that 70% of the participants experienced IPV during the second wave of the COVID-19 pandemic (mid-June 2020 until the end of October 2020). The most common types of violence were emotional or verbal violence (57%) and social or economic violence (45.3%). Physical or sexual violence was also significant (20.8%). This information is supported by data distributed by the “Assistance Center for Victims of Physical and Sexual Assault” of the Arab Society, which reported a 200% increase in IPV during the lockdowns compared with the years 2018--2019 [23,34,35].

Other scholars also reported a high IPV incidence in the Muslim community during the first lockdown [3,10,23]. However, to our knowledge, our study is the first to investigate the prevalence of IPV during the second wave of the COVID-19 pandemic, after citizens had already experienced a first lockdown. After six months of global and local isolation restrictions, women in the Arab minority still suffer from stress, anxiety, depression, and IPV [13]. Some reasons for this could be belonging to an ethnic minority, having fewer available social services (physical boundaries), being afraid to seek social help from professionals (psychological boundaries), and living in low socioeconomic status (i.e., small villages with closed communities, unemployment, and poverty) [21,22].

Traditional cultures, norms, and social attitudes in closed communities emphasize the sanctity of family life but may also make it more difficult for women to report or complain about violence [36]. The IPV incidence in this study (70%) seems even higher than that in other reports from southern Asia and sub-Saharan Africa, with the highest reported prevalence rates of IPV among women aged 15--49 years, ranging from 33%-51% [37]. However, the violence rate is quite high, as reported among women living in conflict-affected areas in rural Uganda (78.5%) [38]. This may raise the possibility that being a minority in a Jewish country with political conflicts might have increased the incidence of IVP, as in Uganda. This point needs further investigation.

The Arab population in Israel comprises approximately 22% of all citizens. The Arabs in Israel are divided into four main Arab subgroups: Bedouin Muslims (21%), non-Bedouin Muslims (60%), Christians (10%), and Druze (9%). These subgroups differ socially, culturally, and geographically. In general, Arabs in Israel, Muslims more than other minorities, are in low living status, mostly beneath the poverty level, and are concentrated in the lowest socioeconomic echelons, with high rates of unemployment. Most Arabs reside in villages, and their residential areas are usually underdeveloped in terms of infrastructure and options for employment [39]. Families and clans are the central units of Arab society and have a collective identity and patriarchal and patrilineal social structure. In addition, there are notions of family honor and female-related honor. Although social, political, educational and economical changes have gradually occurred in the Arab community in Israel during the contemporary years, the social structure of the extended family (“Hamula”) along with the traditional patriarchal culture have kept their major role in affecting the daily life [40,41].

The second wave of the COVID-19 pandemic brought Arab society to a difficult economic situation [13]. This study revealed that at least one partner was on unpaid leave among approximately 43% of the couples, and most participants who were usually employed outside the home (approximately 78% of the women and 82% of their partners) were forced to stay at home during the pandemic (approximately 36% of the women and 25% of their partners). Seven percent of the variance in IPV was explained by demographic characteristics (i.e., caring for more children and coping with lower levels of income)**.** Women who cared for more children reported higher levels of IPV. Muslim women reported greater IPV than Christian women did, as Christians in Israel have higher income and higher academic education, which are positively correlated with lower levels of IPV [41,42].

The current findings are in line with the literature. Imposed social isolation, social distancing, and economic crisis are known risk factors for social, economic, and psychological consequences that may lead to IPV [6,9,32]. The UN Women’s Organization [43] reported that the impact of COVID-19 on violence against women was manifested through increased appeals to emergency centers, women’s shelters, and the police. For many women, quarantine and social isolation increase the burden of household chores and caring for children at home, especially because the children do not regularly attend day-cares, kindergartens, or schools [3]. In our study, we found a positive correlation between the number of children and IPV (Table 4).

The present research revealed that approximately 91% of the women had experienced some degree of violence, which in turn had consequences for at least one domain of mental health-related variables (MHRVs), namely, anxiety, stress, or depression, and approximately 74% of them reported the comorbidity of at least two characteristics of MHRVs. The main characteristic that had significant positive correlations with MHRV was religiosity, as religious women reported higher levels of MHRV than secular, partly religious women did and were Muslim women. High income and academic education were correlated with lower levels of MHRV. We do not have a confident explanation for the positive correlation between IPV and the level of religiosity. However, some studies have shown lower education levels among religious Muslims than among other religions [44], especially among Muslim religious women [45]. This may explain why we found a lower rate of IPV among educated women.

These findings are in line with those of other studies conducted before and during the early stages of the pandemic; however, the recent findings are greater than those of previous studies, where slightly more than a quarter of participants experienced moderate to severe levels of stress and depression [46,47]). One of our explanations may be that the prolonged period under the restrictions, three months during the first COVID-19 wave and now a second lockdown, might have increased the risk for anxiety and depression [48]. Some studies have shown that women are more vulnerable to heightened psychological distress, anxiety, and depression, which have been observed more frequently during the COVID-19 pandemic [46].

In the present study, all types of IPV contributed positively and significantly to stress and anxiety and hence to depression (Fig 1). Women who have experienced violence and abuse are at increased risk of poor health outcomes in a variety of areas [49]. Compared with other types of damage, psychological damage is associated with significantly higher rates of anxiety, depression, and posttraumatic disorders [50,51]. Their children are also at risk of both direct harm and indirect harm because of the poor function of both spouses, the abusers, and the victims [50,52].

IPV is a serious and preventable public health problem that affects millions of people. While lockdowns are designed to protect people from the disease, for some women, the lockdowns resulted in changes in their living circumstances that were associated with negative social and psychological consequences during the lockdown period. Addressing known risk factors to increase awareness of prevention strategies is essential [53–55].

Minimizing IPV can significantly reduce negative mental and health-related experiences such as anxiety, stress, and depression. IPV can lead to a life-threatening situation; similarly, anxiety and depression are considered serious illnesses. Carrying out interventions to reduce IPV will achieve two results: improving women’s security in their homes and reducing depression and anxiety.

These findings may be highly important in devising intervention programs to reduce IPV and encourage a mental health intervention strategy as a follow-up to COVID-19. There are differences in IPV, anxiety, and depression prevalence across cultures Therefore, culturally appropriate and targeted interventions should be implemented, and limited resources should be directed toward at-risk populations.

### Policy implications

The findings also have implications for policy, practice, and social change. At the policy level, existing Arabic-language mental health, social work, nursing, and IPV-related services should be strengthened, better resourced, and made more accessible within Arab communities. At the practice level, discreet and accessible help-seeking pathways should be developed, including Arabic-language digital tools or applications that allow women to obtain information, report IPV, and seek assistance anonymously, particularly during lockdowns or periods in which access to face-to-face services is limited. At the social change level, efforts should focus on reducing stigma and fear of disclosure by using culturally appropriate outreach, targeted social media campaigns, and collaboration with trusted community figures, including religious leaders such as imams and priests. Such strategies may help improve service access and uptake among women who are reluctant to seek formal help because of shame, social pressure, or fear of exclusion.

In conclusion, these results provide important information for identifying factors related to the mental health of Arab women of childbearing age, the prevalence of IPV, and the relationships among these factors. The findings may also be useful in tailoring mental health interventions that are culturally adapted to treat the posttraumatic nature of distress and training health professionals.

### Research limitations

This study has several limitations due to the sampling technique and design. The voluntary recruitment of Facebook may have introduced a bias of choice only for people who are active on Facebook via internet access or smartphones. In addition, the use of online snowball sampling through Facebook and WhatsApp may have introduced further selection bias, as women with lower educational attainment, a larger number of children, stronger religious observance, or more restrictive household circumstances may have been less likely to participate. Therefore, the relatively high proportion of academically educated women in the present sample may have led to an underestimation of the burden of IPV and psychological distress among more disadvantaged subgroups. Moreover, although 55% of respondents reported living in urban localities, a considerable proportion of the Arab population in Israel resides in rural localities and villages. The underrepresentation of rural Arab women may have underestimated the hardships faced by this subgroup, including limited access to support services, greater social isolation, and stronger barriers to disclosure during the pandemic. Self-reports may have also led to bias for women under high stress and vulnerability, following a few months under restrictions. Therefore, future studies should emphasize and use various tools, such as observations or semi-structured interviews, which may be suitable for examining more complex aspects of IPV and emotional aspects during times of severe health crises. Although the present study did not include longitudinal data from the same participants during the first wave, the findings should be interpreted in light of the cumulative burden of the second wave. By this stage of the pandemic, many families had already experienced several months of uncertainty, repeated restrictions, school closures, economic instability, and reduced access to social and professional support systems. These prolonged stressors may have intensified conflict within households and increased women’s exposure to controlling or violent partners. For Arab women living in close-knit communities, the impact of these stressors may have been further amplified by limited privacy, fear of social stigma, and barriers to disclosing IPV or seeking help. Thus, the high rates of IPV, anxiety, stress, and depression observed in the present study may reflect not only the immediate effects of lockdown, but also the cumulative social, economic, and psychological consequences of the pandemic’s second wave. This study has used a cross-sectional design, which lacks the temporal precedence aspect of the mediation analysis, and thus does not allow for causal conclusions. Future longitudinal designs are advised, in order to capture the causal nature of the mediation analysis. Furthermore, our findings suggest a high prevalence of IPV during the pandemic; however, we cannot conclusively say that the prevalence was solely due to the pandemic and that no other stressors were controlled for in the study.

## Abbreviations

COVID-19: Coronavirus disease 2019
IPV: intimate partner violence
MDD: major depressive disorder
MHRV: mental health-related variable
PHQ: Patient Health Questionnaire
PSS: Perceived Stress Scale.
